# Selective Permeabilization of the Blood–Brain Barrier at Sites of Metastasis

**DOI:** 10.1093/jnci/djt276

**Published:** 2013-10-09

**Authors:** John J. Connell, Grégoire Chatain, Bart Cornelissen, Katherine A. Vallis, Alastair Hamilton, Len Seymour, Daniel C. Anthony, Nicola R. Sibson

**Affiliations:** **Affiliations of authors:**CRUK/MRC Gray Institute for Radiation Oncology and Biology, Churchill Hospital, Oxford, UK (JJC, GC, BC, KAV, AH, NRS); Department of Pharmacology (JJC, AH, DCA) and Department of Oncology (JJC, GC, BC, KAV, AH, LS, NRS), University of Oxford, Oxford, UK.

## Abstract

**Background:**

Effective chemotherapeutics for primary systemic tumors have limited access to brain metastases because of the blood–brain barrier (BBB). The aim of this study was to develop a strategy for specifically permeabilizing the BBB at sites of cerebral metastases.

**Methods:**

BALB/c mice were injected intracardially to induce brain metastases. After metastasis induction, either tumor necrosis factor (TNF) or lymphotoxin (LT) was administered intravenously, and 2 to 24 hours later gadolinium- diethylenetriaminepentaacetic acid, horseradish peroxidase, or radiolabeled trastuzumab (^111^In-BnDTPA-Tz) was injected intravenously. BBB permeability was assessed in vivo using gadolinium-enhanced T_1_-weighted magnetic resonance imaging and confirmed histochemically. Brain uptake of ^111^In-BnDTPA-Tz was determined using in vivo single photon emission computed tomography/computed tomography. Endothelial expression of TNF receptors was determined immunohistochemically in both mouse and human brain tissue containing metastases. Group differences were analyzed with one-way analysis of variance followed by post hoc tests, Wilcoxon signed rank test, and Kruskal–Wallis with Dunn’s multiple comparison test. All statistical tests were two-sided.

**Results:**

Localized expression of TNF receptor 1 (TNFR1) was evident on the vascular endothelium associated with brain metastases. Administration of TNF or LT permeabilized the BBB to exogenous tracers selectively at sites of brain metastasis, with peak effect at 6 hours. Metastasis-specific uptake ratio of ^111^In-BnDTPA-Tz was also demonstrated after systemic TNF administration vs control (0.147±0.066 vs 0.001±0.001). Human brain metastases displayed a similar TNF receptor profile compared with the mouse model, with predominantly vascular TNFR1 expression.

**Conclusions:**

These findings describe a new approach to selectively permeabilize the BBB at sites of brain metastases to aid in detection of micrometastases and facilitate tumor-specific access of chemotherapeutic agents. We hypothesize that this permeabilization works primarily though TNFR1 activation and has the potential for clinical translation.

Brain metastases pose a substantial challenge for chemotherapeutic treatment because of the impermeable nature of the blood–brain barrier (BBB), which limits access of drugs and thus prevents accumulation of clinically effective doses at tumor sites. Substances with good penetration of the BBB have limited activity against breast cancer, one of the most common cancers metastasizing to the brain, whereas the most active therapeutics for breast cancer (including doxorubicin and trastuzumab) appear not to reach the central nervous system (CNS) (1) because of their hydrophilic nature. At the same time, the impermeable BBB also prevents early diagnosis of small brain metastases by the current clinical gold-standard method of gadolinium-enhanced magnetic resonance imaging (MRI). This diagnostic approach enables detection of large cerebral metastases and primary brain tumors but only when gross structural abnormalities have developed. Moreover, although BBB compromise may allow limited access of drugs to the tumor in later stages, this BBB permeability is frequently inhomogenous and generally poor around the tumor margin (2). Thus, the late stage of BBB disruption and inhomogeneity across the tumor mean treatment is largely ineffective. Smaller metastases possessing an intact BBB evade both detection and treatment and will inevitably develop into symptomatic tumors.

A number of approaches to transiently circumvent the BBB have been investigated for the delivery of chemotherapeutics to brain tumors [for a review see (3)]. Bradykinin B2 receptor activation by cereport (RPM-7) was the first pharmacological treatment to be shown to transiently modify the BBB in a receptor-mediated manner (4,5) and to increase drug transport into rat and human gliomas. This approach, however, did not improve the efficacy of carboplatin in a phase II trial in glioma patients at the dose used because of the dose-limiting side effect of hypotension (6). The efficacy of RMP-7 in brain metastases has not been investigated. Alternatively, intravenous infusion of the hyperosmotic agent mannitol has been shown to globally induce endothelial cell shrinkage and tight junction separation and has been proposed as a means of transiently providing access to cerebral tumors (7). This approach has been performed in humans and has been shown to cause BBB disruption. However, the lack of specificity for tumor sites is a serious confounder with regard to healthy brain tissue, whereas the short working window limits therapeutic efficacy (8). Alternatively, ultrasound-mediated focused BBB disruption is a promising technique but relies on prior knowledge of metastatic sites (9). Thus, further work in this area is critical if brain metastases are to be detected and treated effectively.

Preclinical studies aimed at increasing drug delivery to systemic tumors have demonstrated the ability of an intravenous bolus dose of recombinant human tumor necrosis factor (TNF), a proinflammatory cytokine, to disrupt endothelial tight junctions in the tumor vasculature through the RhoA/Rho kinase pathway (10). This approach was shown to enhance the permeability of tumor vasculature and to facilitate virus particle delivery to a solid subcutaneous xenograft EL4 lymphoma model in mice. TNF has two endogenous receptors (TNFR1 and TNFR2), which mediate endothelial cytoskeletal reorganization and destabilization of interendothelial adhesion complexes (11). Although their activation is generally associated with pathophysiological processes, the effect of TNF receptor activation in controlled low-dose administration may be beneficial. However, the normal adult brain microvasculature, unlike peripheral blood vessels, is known to be resistant to the permeabilizing effects of cytokines (12). This resistance can be modified by a number of factors, and in previous work we have shown that microinjection of TNF into the rat brain can cause focal, but delayed, disruption of the BBB in association with a focal inflammatory response (13). Our recent work has shown that the early phases of metastasis development in the brain are associated with a strong inflammatory response and activation of the cerebral endothelium (14,15).

Based on the above findings, we hypothesized that cerebral metastases may provide a unique environment for TNF receptor activation on the associated vasculature and that this might yield a target for specific and local opening of the tumor-associated BBB. The primary aims of this study, therefore, were to determine whether TNFR1 and TNFR2 are expressed on metastasis-associated vasculature, and to determine whether intravenous administration of TNF, or its endogenous analogue lymphotoxin (LT), can permeabilize the BBB specifically at sites of cerebral metastasis throughout the brain to an extent that allows entry of 1) diagnostic imaging agents and 2) a clinically relevant anticancer drug.

## Methods

### Brain Metastasis Model

Animal procedures were carried out in accordance with the UK Animals (Scientific Procedures) Act 1986 and with local ethical committee approval. Female BALB/c mice (aged 6–8 weeks; 19.1g (±1.6); Charles River, Margate, UK) were injected in the left ventricle of the heart with 10000 4T1–green fluorescent protein (GFP) (16) murine mammary carcinoma cells in 100 µL of phosphate-buffered saline using ultrasound guidance (VEVO 770; Visualsonics, Amsterdam, The Netherlands) under general anesthesia (1.5%–3.0% isoflurane in oxygen).

### Immunohistochemistry for TNF Receptors

Brain tissue sections from mice injected with 4T1-GFP cells were assessed immunohistochemically for TNFR1 and TNFR2 expression, and colocalization with glucose transporter 1 (Glut-1) was determined using immunofluorescence. Six cases of human brain metastasis and one control case of noncancerous neuroinflammatory disease were examined immunohistochemically for TNFR1 and TNFR2 expression. See the Supplementary Methods (available online) for details of immunohistochemistry and immunofluorescence.

### Histological Assessment of BBB Permeabilization by Cytokine Administration

At 13 days postmetastasis induction in the 4T1-GFP model, mice were intravenously injected with 100 µL saline containing doses from 1 to 5 μg per mouse of either recombinant mouse TNF (Peprotec, London, UK) or recombinant mouse lymphotoxin (R&D Systems, Oxford, UK) or saline as control. Two to 24 hours later, mice were intravenously injected with of 100 µL of type II horseradish peroxidase (44kDa) (HRP; 300 units; SigmaAldrich, Dorset, UK) and killed by transcardial perfusion fixation. Brains were excised and prepared for histology. Slides were stained using a modified Hanker–Yates method and counterstained using cresyl violet. Brown staining indicating permeation of HRP was assessed blindly, and a percentage of positive metastases for each group was calculated. See Supplementary Methods (available online) for details of full experimental protocol and histology.

### In Vivo Assessment of BBB Permeability: MRI

BALB/c mice injected with 4T1-GFP cells (n = 3 per group) were anesthetized and positioned in a quadrature birdcage coil. Respiration was monitored throughout, and body temperature was maintained at 37°C. MRI data were acquired using a 7 T horizontal-bore magnet with a Varian Inova spectrometer (Varian, Santa Clara, CA). Before and 2 hours after the 3-μg intravenous injection of TNF or LT, a set of 10 serial T_1_-weighted images were acquired using a spin-echo sequence both before and 5 minutes after intravenous injection of 30 µL of gadolinium-diethylenetriaminepentaacetic acid (Gd-DTPA, 590Da; Omniscan, GE Healthcare, Little Chalfont, UK) to assess BBB permeability. Regions of interest (ROIs) containing metastases (confirmed histologically) and contralateral same-slice nontumor ROIs were segmented using VnmrJ (Varian) on post-Gd images. The mean signal intensity values of the ROIs were quantitated before and after LT or TNF administration (ROI n = 14 total). See Supplementary Methods (available online) for details of sequence parameters.

### In Vivo Assessment of BBB Permeability: Single Photon Emission Computed Tomography/Computed Tomography

Radiolabelled trastuzumab (^111^In-BnDTPA-Tz, 145kDa) was synthesized as previously described (17). Mice injected with 4T1-GFP cells (n = 3 per group) were injected intravenously with 100 μL of saline containing 3 µg of TNF or LT or no cytokine together with 3.7 µg ^111^In-BnDTPA-Tz, 2 hours before single photon emission computed tomography (SPECT) under anesthesia. SPECT/computed tomography (CT) was performed using a nanoSPECT/CT scanner (Bioscan, Washington, DC). Data were reconstructed using InVivoScope (Bioscan) and analyzed using Inveon Research Viewer (Siemens, Erlangen, Germany) and normalized against a muscle ROI to compensate for intersubject variability in radioactivity dose. See Supplementary Methods (available online) for details.

### Validation in Human Breast Carcinoma Brain Metastasis Model

Female SCID mice (aged 6–8 weeks) were intracardially injected (as above) with 10000 MDA231BR-GFP cells (subclone of a metastatic human breast carcinoma that preferentially metastasizes to the brain) (18). Mice (n = 3) were intravenously injected with saline or 3 μg of TNF and perfusion-fixed 21 days after cell injection. This later time point was chosen because these tumors are slower growing than the 4T1-GFP metastases (15). Tissue sections were stained for TNFR1 and TNFR2 (as above).

### Statistical Analyses

BBB breakdown frequency in metastases was analyzed with one-way analysis of variance (ANOVA) followed by a Dunnet post hoc test or a Tukey post hoc test. The difference in ratio of signal intensity at sites shown to contain metastases vs equivalent regions in the contralateral hemisphere was assessed with the Wilcoxon signed rank test. Intracerebral radioactivity differences were analyzed with the Kruskal–Wallis test followed by a Dunn multiple comparison test. All statistical tests were two-tailed and considered statistically significant if P was less than or equal to 0.05.

## Results

Cerebral metastases were evident in all mice injected with 4T1-GFP cells. Mice displayed transient piloerection after TNF injection. Cross sectional areas of metastases at day 13 ranged in size from 500 μm^2^ to 50000 μm^2^.

### Histological Detection of TNF Receptor Expression

**Figure 1.**
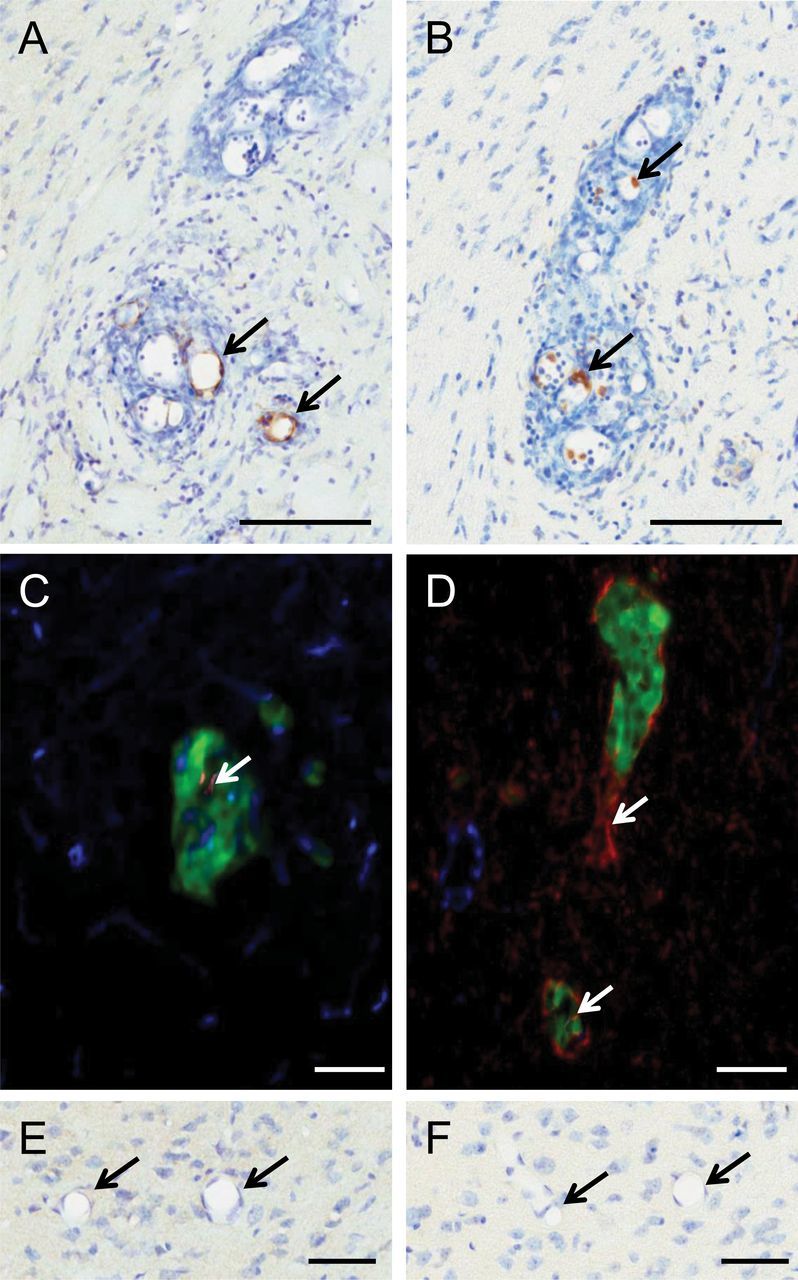
Histological detection of TNF receptors at sites of cerebral metastases in a mouse model. **A** and **C**) Tumor necrosis factor receptor 1 (TNFR1) (A: **brown**; C: **red**) is evident on blood vessels associated with metastases (**arrows**). **B** and **D**) Tumor necrosis factor receptor 2 (TNFR2) (B: **brown**, D: **red**) is not visible on vascular endothelium but appears to colocalize (**arrows**) with intravascular leukocytes (**A**) and infiltrating microglia (**D**). Coronal brain tissue sections are 20 μm. **E** and **F**) Neither receptor is present on nonmetastasis-associated vessels (defined as ≥500 μm from nearest metastasis). **A**, **B**, **E**, and **F**) 3, 3’-diaminobenzidine (DAB) immunohistochemistry with cresyl violet counterstain. **C**) Immunofluorescence for TNFR1 (**red**), glucose transporter 1(Glut-1) (**blue**), and 4T1–green fluorescent protein (GFP) (**green**). **D**) Immunofluorescence for TNFR2 (**red**), Glut-1 (**blue**), and 4T1-GFP (**green**). Scale bars are 100 μm (**A**–**D**) or 50 μm (**E** and **F**).

Expression of TNFR1 was immunohistochemically localized to the vessels associated with metastases in mice injected intracardially with 4T1-GFP cells (Figure 1, A and C, arrows). TNFR1 expression on the vasculature of metastases was confirmed through colocalization of TNFR1 and Glut-1 by immunofluorescence. In contrast, TNFR2 expression, although also increased, was found on intravascular leukocytes within the metastases, but not on the vascular endothelium (Figure1, B and D, arrows). TNFR2 expression was also detected on nonvascular structures with a morphology similar to that of microglia. No TNFR1 or TNFR2 staining was found on normal appearing brain tissue (Figure 1, E and F).

### Histological Detection of BBB Permeabilization

**Figure 2.**
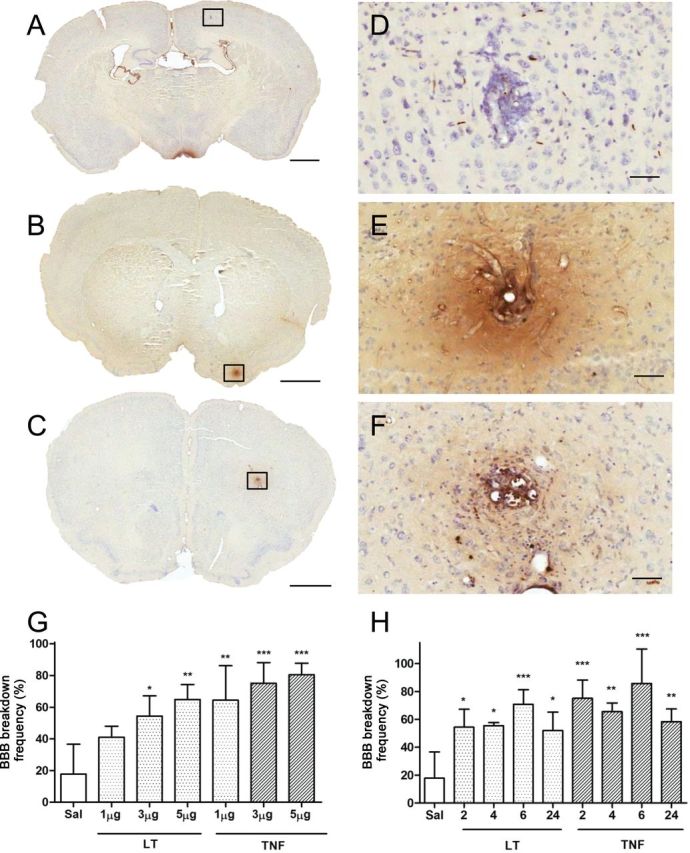
Histological detection of brain metastases and cytokine-induced blood–brain barrier (BBB) breakdown. Photomicrographs of brain metastases from mice injected systemically with horseradish perioxidase (HRP) and saline (**A** and **D**), 3 μg of lymphotoxin (LT) (**B** and **E**), or 3 μg of tumor necrosis factor (TNF) (**C** and **F**). Hanker–Yates histology for HRP detection (**brown**) reveals areas of BBB breakdown. Cresyl violet counterstain. **G**) Dose–response analysis of metastasis-specific BBB breakdown frequency 2 hour after systemic administration of differing doses of LT or TNF. **H**) Temporal analysis of metastasis-specific BBB breakdown frequency after systemic administration of 3 μg of LT or TNF. Data were analyzed blind to treatment group. Scale bars are 1mm (**A**–**C**) or 50 μm (**D**–**F**). Statistical analysis: two-sided 1-way analysis of variance with Dunnet post-hoc test vs saline group (*P < .05, **P < .01, ***P < .005). Error bars represent standard deviation.

After intravenous injection of HRP, BBB permeabilization was defined as brown HRP staining within Hanker–Yates-treated tissue sections at metastases larger than 10 cells. One thousand one hundred and two separate metastatic colonies were blindly analyzed to determine their status with respect to BBB integrity. No BBB breakdown was observed in 4T1-GFP–injected mice at nonmetastatic sites before or after cytokine injection (Figure 2, A and D). TNF (Figure 2, B and E) or LT (Figure 2, C and F) induced HRP staining generally restricted to the metastatic colony, although some metastases displayed minor spread to the surrounding tissue. The frequency with which BBB breakdown was observed in metastases after cytokine treatment was statistically significantly higher than in noncytokine-treated controls (TNF 3 μg: 75.1% ± 13.1% vs. control: 17.8% ± 18.9%) (Figure 2G). The frequency of BBB breakdown in metastases appeared to escalate with dose (1, 3, or 5 μg of LT or TNF), but this did not reach statistical significance (one-way ANOVA with Tukey post-test). The frequency of BBB breakdown after administration of either LT or TNF (3 μg) appeared to peak at 6 hours (Figure 2H). Breakdown was still evident at 24 hours but appeared to be decreasing in both TNF and LT groups. Although this decrease did not reach statistical significance (one-way ANOVA with Tukey post-test), by 72 hours no statistically significant differences were found in incidence of breakdown between TNF and control groups (Supplementary Figure 1A, available online).

### In Vivo MRI Detection of BBB Permeabilization

**Figure 3.**
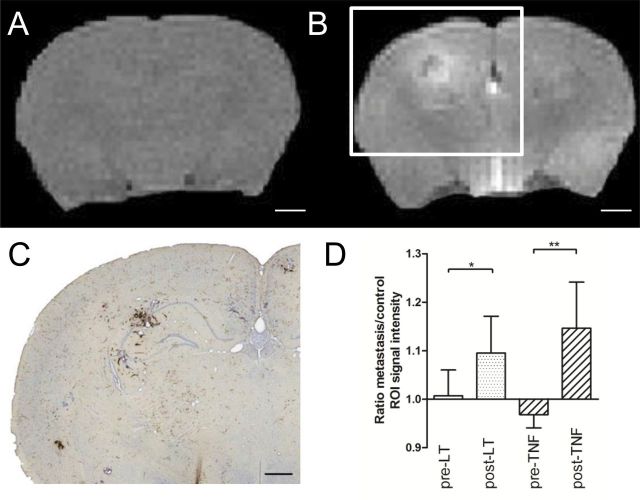
Magnetic resonance imaging detection of cytokine-induced blood–brain barrier (BBB) breakdown. T_1_-weighted images from tumor necrosis factor (TNF)–treated, metastasis-bearing mouse brain before (**A**) and 5 minutes after (**B**) gadolinium-diethylenetriaminepentaacetic acid (Gd-DTPA) injection. Areas of high signal intensity (**white**) infer areas of BBB breakdown and Gd-DTPA entry. **C**) Photomicrograph of Hanker–Yates-stained tumor colony from brain slice corresponding to white box in (**B**). **D**) Region of interest (ROI; n = 14) signal intensities corresponding to histologically verified metastatic sites in lymphotoxin (LT)– and TNF-treated brains were statistically significantly higher than equivalent control ROIs. Signal intensity of metastasis ROI divided by signal intensity of equal ROI on contralateral hemisphere: ratio of 1.0 represents no breakdown. Wilcoxon signed rank test (two-sided): LT (n = 6; *P =* .03), TNF (n = 8; *P* = .008). Scale bars are 1mm (**A** and **B**) or 500 μm (**C**). Statistical analysis: two-sided 1-way analysis of variance with Dunnet post-hoc test vs saline group (*P < .05, **P < .01, ***P < .005). Error bars represent standard deviation.

The T_1_-weighted image of mouse brain (Figure 3A) before contrast agent administration shows no contrast. Extravasation and accumulation of paramagnetic Gd-DTPA reveals the presence of BBB breakdown as hyperintense areas on T_1_-weighted images. After injection of either LT or TNF, several areas of hyperintensity were evident on T_1_-weighted images (Figure 3B) from mice injected with 4T1-GFP cells, which correlated with sites of metastatic colonies found histologically (Figure 3C). The ratio of signal intensity at sites shown to contain metastases vs equivalent regions in the contralateral hemisphere was statistically significantly greater 2 hours after administration of 3 μg of LT (n = 6; *P =* .03) or TNF (n = 8; *P* = .008, two-sided Wilcoxon signed rank test) (Figure 3D).

### In Vivo Detection of Radiolabelled Trastuzumab

**Figure 4.**
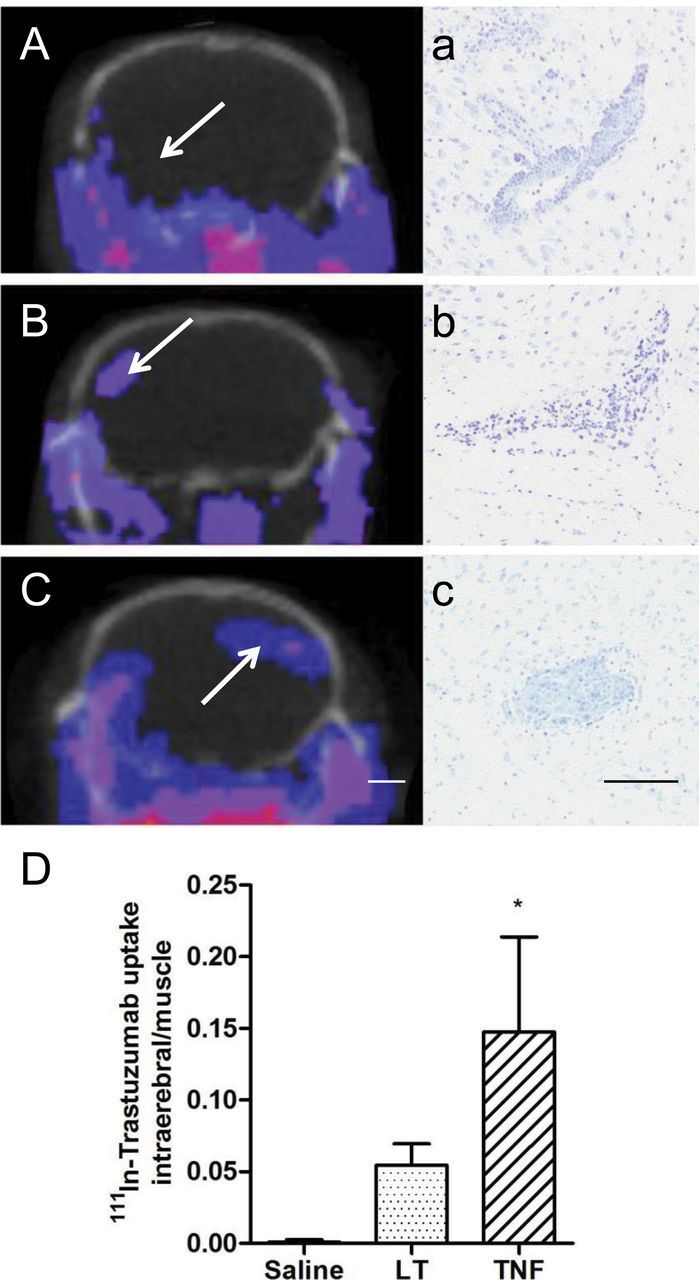
Detection of 111-indium-benzyl diethylenetriaminepentaacetic acid-trastuzumab (^111^In-BnDTPA-Tz) using single photon emission computed tomography/computed tomography (SPECT/CT). Single slice SPECT/CT images showing localization of radiolabeled antibody (**blue-pink**) in mice treated with saline (**A**), 3 μg of lymphotoxin (LT) (**B**), or 3 μg of tumor necrosis factor (TNF) (**C**) (n = 3 each group). Intracerebral SPECT signal reveals accumulation of antibody at sites of BBB breakdown. Regions showing SPECT signal enhancement (**arrows**) were later confirmed to be sites of metastasis with cresyl violet histology (**a**–**c**). Note site of metastasis shown in (**a**) was not detected with SPECT in mouse injected systemically with saline (**A**; **arrow**). **D**) Ratio of radioactivity count from sum of intracerebral regions of interest (ROIs) compared with muscle ROI. Scale bars are 2mm (**A**–**C**) or 100 μm (**a**–**c**). Statistical analysis: two-sided Kruskal–Wallis with Dunn multiple comparison test (*P < .05). Error bars represent standard deviation.

^111^In-BnDTPA-Tz was administered to determine whether cytokine-mediated permeability changes facilitated trastuzumab delivery to metastases. Antibody was excluded from the brain at sites of metastasis when injected systemically with saline (Figure 4A), as well as in brains of mice injected with 4T1-GFP cells at nontumor sites. In contrast, after systemic injection of either LT (Figure 4B) or TNF (Figure 4C), isolated regions of increased SPECT signal were evident within the brain, which were associated histologically with sites of metastases (Figure 4, A–C). Intracerebral radioactivity was statistically significantly higher in mice treated with TNF than controls (0.147±0.066 vs 0.001±0.001; *P* < .05, Kruskal-Wallis with Dunn’s multiple comparison test) (Figure 4D).

### Validation in Human Breast Carcinoma Brain Metastasis Model

To further confirm the effects of TNF on metastasis permeabilization, the MDA231BR-GFP model was used. The frequency of BBB breakdown in metastases after cytokine treatment was statistically significantly higher than in noncytokine-treated controls (*P* < .05, unpaired *t* test) (Supplementary Figure 2A, available online), and a similar expression profile of TNFR1 and TNFR2 was observed as for the 4T1-GFP model (Supplementary Figure 2, B and C, available online).

### TNF Receptor Expression in Human Brain Metastasis Tissue

**Figure 5.**
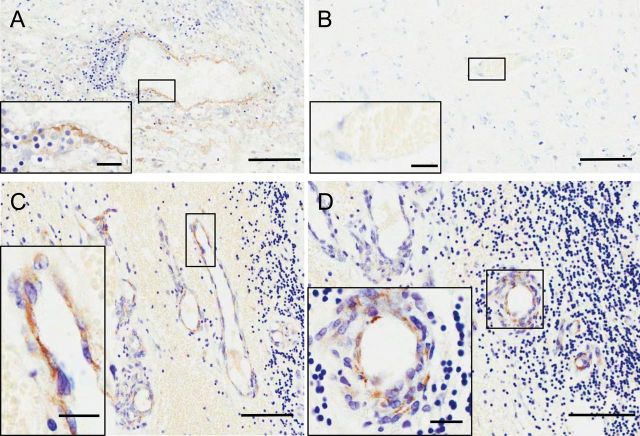
Endothelial TNFR1 expression in human brain metastasis. Large insets are magnified images of boxed areas. **A**) Positive control section showing tumor necrosis factor receptor 1 (TNFR1) expression (**brown**) on a brain vessel adjacent to nonspecific inflammation. **B**) Normal brain tissue showing minimal TNFR1 reaction in cortical vessel. **C** and **D**) Staining for TNFR1 on brain parenchymal endothelial cells at sites of metastasis. Scale bars are 100 μm (**A**–**D**) or 20 μm (**insets**).

To determine whether this local cytokine-mediated permeabilization of the BBB in brain metastasis may be clinically translatable, six biopsies from brain metastasis were stained for TNFR1 and TNFR2. Endothelial TNFR1 staining was confirmed in a control human brain sample with nonspecific inflammation (Figure 5A), whereas immunoreactivity was negligible in control brain tissue (Figure 5B). In contrast, positive TNFR1 immunoreactivity was found on the endothelium of metastasis-associated vessels in five of six of the biopsies analyzed (Figure 5, C and D). Additionally, TNFR2 was variably found on neurons local to the metastasis, leukocytes, and intravascular neutrophils (Supplementary Figure 3, available online).

## Discussion

We have described a new approach to permeabilize the vasculature of brain metastases that is based specifically on the stromal vascular phenotype of these tumors, with a view to enhancing detection and improving the delivery of chemotherapeutic agents. The 4T1-GFP mouse model of brain metastasis was used to generate micrometastases in the brain, which, at the age used here, rarely present with a permeable BBB. To ensure that all metastases within the brain were targeted, the cytokines LT or TNF were injected intravenously and were found to induce permeabilization of the BBB specifically at the sites of brain metastases, as revealed both ex vivo through histology (HRP) and in vivo through contrast-enhanced MRI (Gd-DTPA). We further replicated these findings in a second model of human-derived brain metastasis and again found that systemically administered TNF increased permeability focally at tumor sites. With this method, the commonly used treatment for breast cancer, trastuzumab (145kDa) (19), which is excluded from the brain under normal conditions, was successfully delivered selectively to metastatic colonies in the brain as revealed by SPECT/CT imaging. We suggest that this permeabilization works primarily though the activation of TNFR1, which was found specifically on the vascular endothelium of vessels closely associated with brain metastases in both mouse models studied and also in human brain metastasis tissue. Our findings suggest that the degree of permeabilization would be sufficient for diagnostically and therapeutically relevant molecules to gain access to the tumor site.

Brain metastases in mice showed increased expression of both receptors for LT and TNF (TNFR1 and TNFR2) before any cytokine treatment. TNFR1 expression was exclusively found on blood vessels within the metastases and colocalized with the endothelial marker Glut-1. These findings suggest the potential for systemic administration of either TNF or LT to alter BBB function that is likely to be through activation of TNFR1. The downstream effects of endothelial TNFR1 stimulation include alterations to cytoskeletal proteins (20–22), cell–cell adhesion molecules (23,24), and changes to paracellular permeability of the BBB (25,26). Moreover, in vitro studies have shown that TNF can increase permeability of endothelial cells (27,28).

At sites of brain metastasis, expression of TNFR2 was also clearly increased but restricted to what appear to be recruited intravascular leukocytes and parenchymal microglia. This receptor expression pattern is in accord with previous studies reporting TNFR2 expression by immune system cells (29). Given this and the fact that soluble TNF cannot induce full activation of TNFR2 (30), it appears likely that this receptor plays a lesser or secondary role in the cytokine-induced permeabilization observed in this work.

After treatment with either cytokine, the intravenous tracer HRP was found within metastases in the brain, indicating compromise of the BBB. Increasing cytokine dose was accompanied by an increase in the frequency of metastases exhibiting a permeable BBB. Breakdown of the BBB was specific and local to the sites of brain metastases, and no other permeabilization of the BBB was observed in normal brain tissue. These findings support the concept that upregulation of TNFR1 on tumor-associated vessels alone enables specific permeabilization of the tumor vasculature in response to systemic LT or TNF. This effect was maintained for all of the time points studied here. The greatest number of HRP-positive metastases was evident at 6 hours, whereas the lower number of positive metastases at the 24-hour time point suggests that this is an extended but transient opening of the BBB. The concentration of the administered cytokine rapidly returns to baseline levels (31), which supports the concept that endothelial TNFR1 signaling pathways, subsequent to receptor activation, give rise to reorganization of the junctional complexes beyond the clearance time of the cytokine bolus. The length of time of the increase in permeability suggests that the vasculature may be structurally compromised by the treatment, and TNF is known to be cytotoxic to endothelial cells (32). However, we found no evidence of hemorrhage, suggesting that frank vascular injury is not the mechanism for permeabilization (Supplementary Figure 1, B–D, available online).

LT elicited a less marked response than TNF at each dose and time point, which is in agreement with previous studies that describe a less stable complex formation of lymphotoxin with TNFR1 compared with TNF, thus inducing a lower signaling capability (33).

Numerous local hyperintensities were seen on post-Gd-DTPA T_1_-weighted images after treatment with LT or TNF. Although these regions were associated with sites of metastasis shown histologically, other sites of metastasis were not detectable with MRI. The apparent lack of permeability may be because of the limited resolution of our in vivo scans (approximately 160 μm), giving a higher size detection threshold than the histological method (approximately 1 μm). Alternatively, it is possible that the micrometastases must show a particular growth pattern or cell recruitment profile before TNFR1 expression is sufficient to allow permeabilization. Nevertheless, even metastases of approximately 50 μm diameter showed sensitivity to cytokine-induced permeabilization, which is considerably smaller than those currently detectable clinically owing to natural BBB permeability (0.5–1.0cm diameter) (34,35).

Trastuzumab is a monoclonal antibody that is active against *HER2*-overexpressing breast cancer, leading to reduced breast tumor burden and increased patient survival (36). Extravasation of circulating antibodies and binding to the Her2 receptor leads to a number of antitumor actions [reviewed in (37)]. However, trastuzumab is ineffective in treating brain metastases, in part because of restricted access to the Her2 receptor once the metastatic tumor cells are sequestered on the brain side of the BBB, because it does not effectively cross the BBB (1,38,39). Similarly, in the 4T1-GFP model used here, trastuzumab was excluded from brain metastases in saline-treated controls. However, in mice treated systemically with LT or TNF, the antibody was found to permeate the BBB and accumulate within the brain to a level detectable by SPECT imaging. Metastases were present at all sites of intracerebral SPECT signal but were much smaller than the volume of signal presenting on the SPECT/CT image. Thus, although accurately displaying the amount of radioactivity, the partial volume effect and potential spread of antibody from point of delivery may over-represent the metastasis size. As with our MRI studies, some metastases were present in the brain that did not appear to accumulate radiolabelled antibody. Again, this may reflect the low spatial resolution of SPECT detection (approximately 1mm) precluding detection of small micrometastases. Once again the metastases that were detected and thus exposed to the therapeutic compound were well below the detection threshold currently possible clinically (34,35) and clearly represent metastases that would not otherwise be accessible to the therapeutic agent. Thus, this approach confers a substantial advantage for treatment of early brain metastases when efficacy may be greatly enhanced.

Six cases of human brain metastasis were analyzed, with different primary tumor origins. Because these metastases had been clinically detected and excised, they were substantially larger than those in the mouse model. Some degree of necrosis and mucus deposition was present, yet critically a similar expression profile for TNFR1 and TNFR2 was seen. In particular, TNFR1 was heterogeneously expressed on metastasis-associated vessels and was not found in nonmetastasis brain tissue. These findings suggest that a similar selectivity in response to a TNF/LT dose could be elicited in human brain metastases. Additionally, TNFR1 and TNFR2 expression was apparent on other cell types in the close vicinity of the metastasis. However, it is less likely that the nonendothelial TNF receptors would contribute substantially to the endothelial changes in the BBB integrity.

Previous work involving bradykinin analogs in glioma models demonstrated a peak of increased drug delivery after 15 minutes of RMP-7 infusion (40), a size limitation for access to the brain of 1 kDa–sized molecules (41) and dose-limiting side-effects. Interestingly, it has been suggested that a possible mechanism of action of bradykinin involves the accelerated release of TNF (42). The approach described here of a direct systemic administration of TNF or its endogenous analog LT may have three major advantages over the use of bradykinin. First, the window of permeability shown here appears to peak 6 hours after cytokine administration and to be maintained to at least 24 hours. This extended window of permeability would increase the opportunity for intravenous drugs to bind to their targets. Second, entry of a range of molecules was facilitated with this approach, from gadolinium-DTPA (590Da) to HRP (44kDa) and up to the therapeutic monoclonal antibody, trastuzumab (approximately 148kDa), suggesting that drug size may not be prohibitive. Finally, although conscious of the potential concerns over TNF toxicity, the human equivalent of the range of doses used in the mouse model here would elicit the desired response within the maximum tolerable dose [150 μg/m^2^ (43)]. The toxicity profile of lymphotoxin has been reported to be substantially below that of TNF at similar levels of antitumor activity (44). Further, at the dose of TNF used here, no statistically significant differences were found between any of the groups for markers of the acute phase response and hepatoxicity (Supplementary Methods and Supplementary Figure 4, A–E, available online). It is also worth noting the very short half-life of TNF within the circulation [2.8min (45)], and we would, therefore, expect any effects of the cytokine bolus on the biology of the tumor to be minimal. However, to test this possibility we used an adenovirus to induce prolonged expression of systemic TNF and found no statistically significant difference in the number or volume of metastases within the brain compared with animals injected with either the equivalent null adenovirus or no virus (Supplementary Methods and Supplementary Figure 5, A and B, available online). There are also known interactions between HER2 and TNF signaling pathways^46^ but, once again, we would expect the short half-life of the cytokine bolus to minimize any unpredictable interactions. Moreover, given the proposed mechanism of TNF action through TNFR1, the use of selective TNFR1 agonists [eg, htr-9–specific TNFR1 antibody (15)] or selective TNF muteins [eg, LK805 (47)] may provide a more targeted approach with fewer potential side effects.

This study also had some limitations. For example, although we have shown that TNF will increase local permeability in two mouse models of metastasis and the pattern of receptor expression is similar in human metastatic cancer, species-specific alterations in TNFR1 and TNFR2 signaling pathways may give rise to different effects in human. Our findings now need to be validated in a limited clinical trial. Additionally, in our models there is no primary tumor, which may alter the systemic inflammatory phenotype. However, it is often the case that the primary tumor has been successfully removed in individuals with brain metastases; hence our models may actually have good clinical relevance in terms of disease burden.

The work presented here shows a novel approach to facilitating the delivery of therapeutic and diagnostic agents to cerebral metastases by exploiting a previously unknown phenotype of the vasculature of brain metastases. Critically, even small metastases (approximately 200-fold smaller than those currently detectable in the clinic) are targeted with this approach. The identification of a similar vascular phenotype in human brain metastasis tissue indicates the potential for clinical translation. This work has demonstrated that cytokine-enhanced drug delivery to brain metastases is possible and that this strategy may be critically important for the detection and treatment of brain metastases clinically.

## Funding

This work was funded by Cancer Research UK (grant C5255/A12678) and the Medical Research Council (UK) (DPhil studentship [JC] and Strategic Skills Award G1000402).

## Supplementary Material

Supplementary Data

## Notes

N.R. Sibson supervised all of the experiments and data acquisition, and jointly conceived the methodology with D.C. Anthony, L. Seymour, and J.J. Connell. J.J. Connell and G. Chatain performed the experiments. B. Cornelissen and K.A. Vallis assisted with the SPECT/CT experiment. A. Hamilton performed the adenovirus experiments.

We would like to thank Dr Alexandre A. Khrapitchev, Dr Sébastien Serres, and Dr Manuel Sarmiento Soto for technical assistance; Dr Sébastien Serres for kindly providing rat brain tissue; and Dr Olaf Ansorge for access to samples of human brain metastases.
