# Supplementary material for: Selective Permeabilization of the Blood–Brain Barrier at Sites of Metastasis
Source: J Natl Cancer Inst. 2013 Oct 9;105(21):1634–43. doi: 10.1093/jnci/djt276 (PMC3818170; doi:10.1093/jnci/djt276)
Supplement: Supplementary Data [file supp_105_21_1634__index.html]

Selective Permeabilization of the Blood–Brain Barrier at Sites of Metastasis — Selective Permeabilization of the Blood–Brain Barrier at Sites of Metastasis — Supplementary Data 

# Selective Permeabilization of the Blood–Brain Barrier at Sites of Metastasis

## Supplementary Data

Data files

**Files in this Data Supplement:**

- Supplementary Data - Supplementary Data
- Supplementary Data - Supplementary Data
