# Supplementary material for: Selective Permeabilization of the Blood–Brain Barrier at Sites of Metastasis
Source: J Natl Cancer Inst. 2013 Oct 9;105(21):1634–43. doi: 10.1093/jnci/djt276 (PMC3818170; doi:10.1093/jnci/djt276)
Supplement: Supplementary Data [file supp_djt276_jnci_JNCI_13_0159_s01.pdf]

Supplementary Methods

Immunohistochemistry and immunofluorescence for localization of TNF receptors

20 µm frozen brain tissue sections were collected on glass slides and heated in citrate buffer (10 mM sodium citrate, 0.05% Tween20, pH 6.0) for 30 minutes for antigen retrieval. Tissue was quenched for 30 min with 1% H_2O_2 in methanol and blocked using 10% normal horse serum for 1 h. Serial tissue sections were exposed to primary antibodies for 16 h at 4 °C (TNFR1, goat anti-mouse, 1:50, R&D Systems or TNFR2, goat anti-mouse, 1:100, R&D Systems) and, subsequently, to secondary antibody for 1 h at room temperature (biotinylated horse anti-goat, 1:100, Vector Laboratories), followed by DAB staining and counterstaining, mounting and imaging as described above.

Human brain tissue sections (4 µm formalin fixed paraffin-embedded) were dewaxed in xylene and rehydrated in decreasing concentrations of ethanol in water and then stained and processed as above (TNFR1, rabbit anti-human AbCam, 1:500; TNFR2, rabbit anti-human AbCam, 1:10).

Other tissue sections were quenched with 1% H_2O_2 in PBS, streptavidin and biotin blocked (SP-2002, Vector Laboratories) and incubated with Tris-NaCl blocking buffer (TNB, PerkinElmer). Sections were subsequently incubated for 16 h at 4°C with the appropriate antibodies (Glut-1, 1:300, AbCam with either TNFR1, 1:200, R&D Systems or TNFR2, 1:200, R&D Systems), rinsed with PBS and incubated with the appropriate secondary antibody in TNB for 30 min. Sections were washed with PBS, incubated with streptavidin-HRP (PerkinElmer; 1:200) in TNB for 30 min, washed and incubated for 8 min in the dark with TSA-biotin (PerkinElmer; 1:100) in amplification buffer (PerkinElmer). Slides were washed and incubated with a

streptavidin-Cy3 fluorophore (Invitrogen, 1:300) and AMCA-conjugated secondary antibody (1:150) for 2 hours. Slides were coverslipped using Vectashield mounting medium (Vector Laboratories). Images were acquired using a Leica DM IRBE (Leica, Germany) attached to a camera (Hamamatsu, Japan) and analysed using ImageJ.

Histological assessment of BBB permeabilization by cytokine administration

At 13 days post-metastasis induction, mice were injected via a tail vein with 100 µl saline containing either recombinant mouse tumor necrosis factor (TNF; Peprotec, London, UK) or recombinant mouse lymphotoxin (LT; R&D Systems, Oxford, UK) or saline as control. Effect of cytokine administration was studied at four different time points (2, 4, 6 or 24 h) prior to perfusion-fixation at a 3 µg dose of either TNF or LT (n=3 per group). Subsequently, dose-response was determined using three different doses of TNF or LT (1, 3 or 5 µg) administered 2 h prior to perfusion-fixation (n=3 per group). To identify areas of BBB permeability, mice were injected via a tail vein with 100 µl type II horseradish peroxidise (300 units; SigmaAldrich, Dorset, UK) and transcardially perfused 30 minutes later under terminal anaesthesia with 50 ml 0.9% herparinised saline followed by 50 ml Karnovsky's fixative (1.25% paraformaldehyde; 2.5% glutaraldehyde; 0.1 M phosphate buffer; pH 7.3). Brains were post-fixed for 4 h, cryoprotected for 24 h in 30% sucrose solution, embedded in Tissue-Tek (Sakura, Finetek, Torrance, CA, USA) and frozen in isopentane at -40 °C. Free-floating 20 µm thick coronal brain sections were collected in 0.1 M phosphate buffer and stained using a modified Hanker-Yates method[1], mounted on glass slides (Superforst Plus, Menzel Gläzer, Braunschweig, Germany) and counterstained with 1% cresyl violet acetate (SigmaAldrich). Slides were imaged using ScanScope CS slide scanner (Aperio, Vista, CA, USA) and analysed using ImageScope (Aperio). Statistical analyses were

performed using GraphPad Prism 5 for Windows (GraphPad Software, La Jolla, California, USA). The histological tracer horseradish peroxidase (HRP) is a 44 kDa glycoprotein that is excluded from the neuropil by the BBB based on its molecular size, and is only detected in the brain at sites of incomplete BBB such as the median eminence. Each metastasis was described as either positive or negative for brown staining indicating BBB permeabilization, giving a percentage of positive metastases for each group. Statistical tests used were one-way ANOVA with Dunnet's post-hoc test of each group vs. saline control group.

In vivo assessment of BBB permeability: MRI

10 serial T_1-weighted images (coronal; 1 mm thick; in plane resolution *ca.* 160 µm; acquisition time *ca.* 3 min) were acquired using a spin-echo sequence (TR, 300 ms; TE, 14 ms; FOV, 20 x 20 mm; matrix, 128 x 128; averages, 4; interleaved) both prior to and 5 min after intravenous injection of 30 µl Gadolinium-DTPA (Gd-DTPA; Omniscan, GE Healthcare, Little Chalfont, UK) to assess BBB permeability.

In vivo assessment of BBB permeability: SPECT/CT

Purified isolated trastuzumab (Tz) (Herceptin, Genentech, USA) was conjugated to para-isothiocyanyl-benzyl-diethylenetriaminepentaacetic acid (p-SCN-BnDTPA) (Macrocyclics, Texas, USA) to allow ^{111}In labeling. Excess BnDTPA was removed by size exclusion chromatography using a Sephadex G50 mini-column (Sigma, Mississauga, ON, Canada). Radiolabeling was performed by adding 2 MBq [^{111}In]$InCl_3$ per microgram of antibody (MDS Nordion, Vancouver, BC, Canada) to the BnDTPA-Tz immunoconjugate. The radiochemical yield was satisfactory at 97%, determined by instant thin layer chromatography run in 0.1 M sodium citrate (pH 5.0). Mice were injected intravenously with 100 µl saline containing 3 µg TNF or LT

or no cytokine together with 3.7 µg ^{111}In-BnDTPA-Tz, 2 h prior to single photon emission computed tomography (SPECT) imaging. Anaesthesia was induced with 4% isoflurane in air and maintained at 1.5-2.5% isoflurane. SPECT/CT was performed using a nanoSPECT/CT scanner (Bioscan, Washington, USA) (FOV, 35.2 x 35.2 mm; data matrix, 186 x 186; acquisition time, *ca*. 1 h). Data were reconstructed using InVivoScope (Bioscan) and analysed using Inveon Research Viewer (Siemens).

Effect of TNF on liver function and metastasis growth

Blood was collected and tested for markers of hepatotoxicity from animals 0, 24 and 72h after a bolus i.v. injection of rmTNF (3 µg; n=3 per group) to assess the impact of low dose TNF on liver function. The following assays were performed on EDTA-plasma samples by Sequani Ltd (Ledbury, UK): total protein, glutamate dehydrogenase (GLDH), alkaline phosphatase (ALP), aspartate transaminase (AST), alanine aminotransferase (ALT).

Chronic systemic TNF expression was induced by intravenous administration of a replication deficient adenovirus expressing membrane-bound TNF cDNA[2] 5 days after intracardiac injection of 10^4 4T1-GFP cells. Mice were perfusion-fixed at day 10 after metastasis induction and both number and volume of metastases in the brain were quantified as described previously[3]. Tumor burden was compared with both animals injected systemically at day 5 with a null adenovirus and non-virus injected animals.

References

1. Hanker JS, Yates PE, Metz CB, Rustioni A. A new specific, sensitive and non-carcinogenic reagent for the demonstration of horseradish peroxidase. *Histochem J*. 1977;9(6):789–792.

2. Sibson NR, Blamire AM, Perry VH, Gauldie J, Styles P, Anthony DC. TNF-alpha reduces cerebral blood volume and disrupts tissue homeostasis via an endothelin- and TNFR2-dependent pathway. *Brain J Neurol*. 2002;125(Pt 11):2446–2459.

3. Serres S, Soto MS, Hamilton A, et al. Molecular MRI enables early and sensitive detection of brain metastases. *Proc Natl Acad Sci U S A*. 2012;109(17):6674–6679. doi:10.1073/pnas.1117412109.
