# Supplementary material for: Selective Permeabilization of the Blood–Brain Barrier at Sites of Metastasis
Source: J Natl Cancer Inst. 2013 Oct 9;105(21):1634–43. doi: 10.1093/jnci/djt276 (PMC3818170; doi:10.1093/jnci/djt276)
Supplement: Supplementary Data [file supp_djt276_jnci_JNCI_13_0159_s02.pdf]

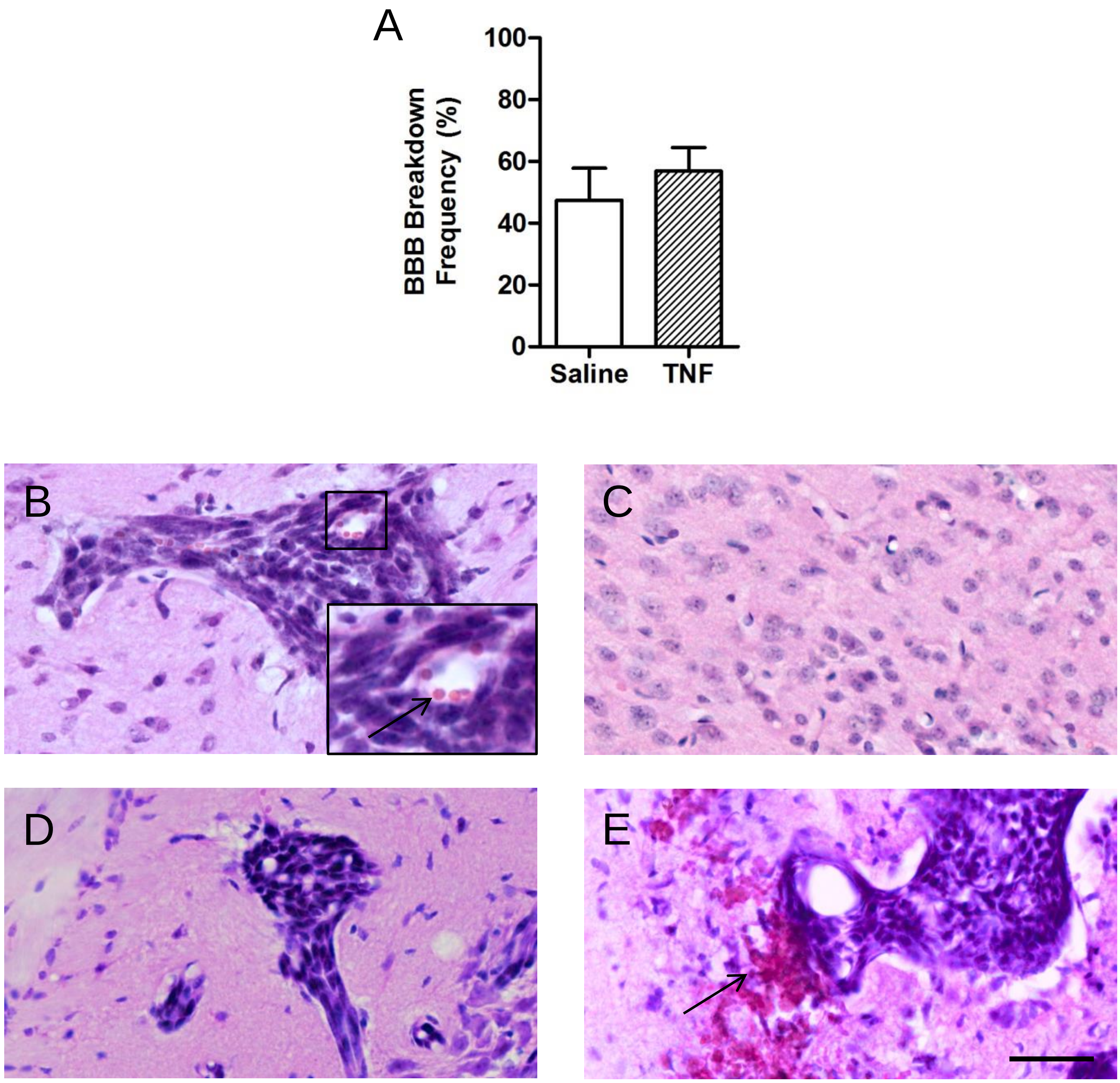

Supplementary Figure 1. (A) Incidence of BBB breakdown at sites of metastasis in BALB/c mice injected intracardially with 4T1-GFP cells 72 hours after intravenous rmTNF or saline administration. (B - D) Photomicrographs showing haematoxylin and eosin staining of brain tissue from mice injected intracardially with 4T1-GFP cells, 72 h after either TNF (B - C) or saline (D) administration (i.v.). No extravascular erythrocyte accumulation was evident either at sites of metastasis (B, D) or in normal parenchymal brain tissue (C). Inset in B shows higher magnification of boxed region; arrows indicate the presence of a small number of intravascular red blood cells. These data suggest that vessel destruction is not the primary underlying cause of the increased vascular permeability following systemic TNF treatment. (E) Photomicrograph showing positive control of frank haemorrhage in a later stage brain metastasis in the rat. Scale bar = 50 μm. Error bars represent standard deviation. Acronyms: BBB (blood-brain barrier), 4T1-GFP (4T1-green fluorescence protein), rmTNF (recombinant mouse tumor necrosis factor), i.v. (intravenous).

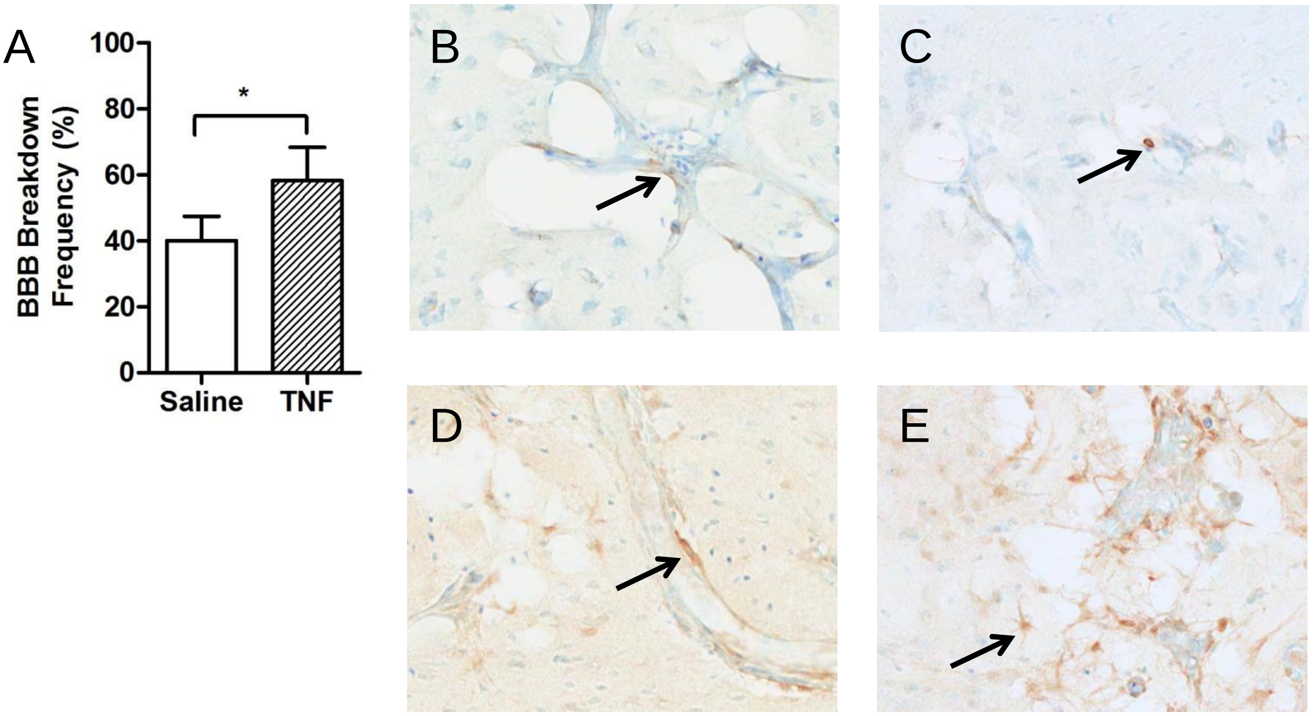

Supplementary Figure 2. (A) Incidence of BBB breakdown in SCID mice 21 days after intracardiac injection of MDA231BR-GFP cells. (B - C) TNFR1 and TNFR2 expression at sites of metastasis 21 days after intracardiac injection of MDA231BR-GFP cells. (D - E) TNFR1 and TNFR2 expression at sites of metastasis 21 days after intracerebral injection of MDA231BR-GFP cells. Acronyms: BBB (blood-brain barrier), SCID (severe combined immunodeficiency), TNFR1/2 (tumor necrosis factor receptor 1/2).

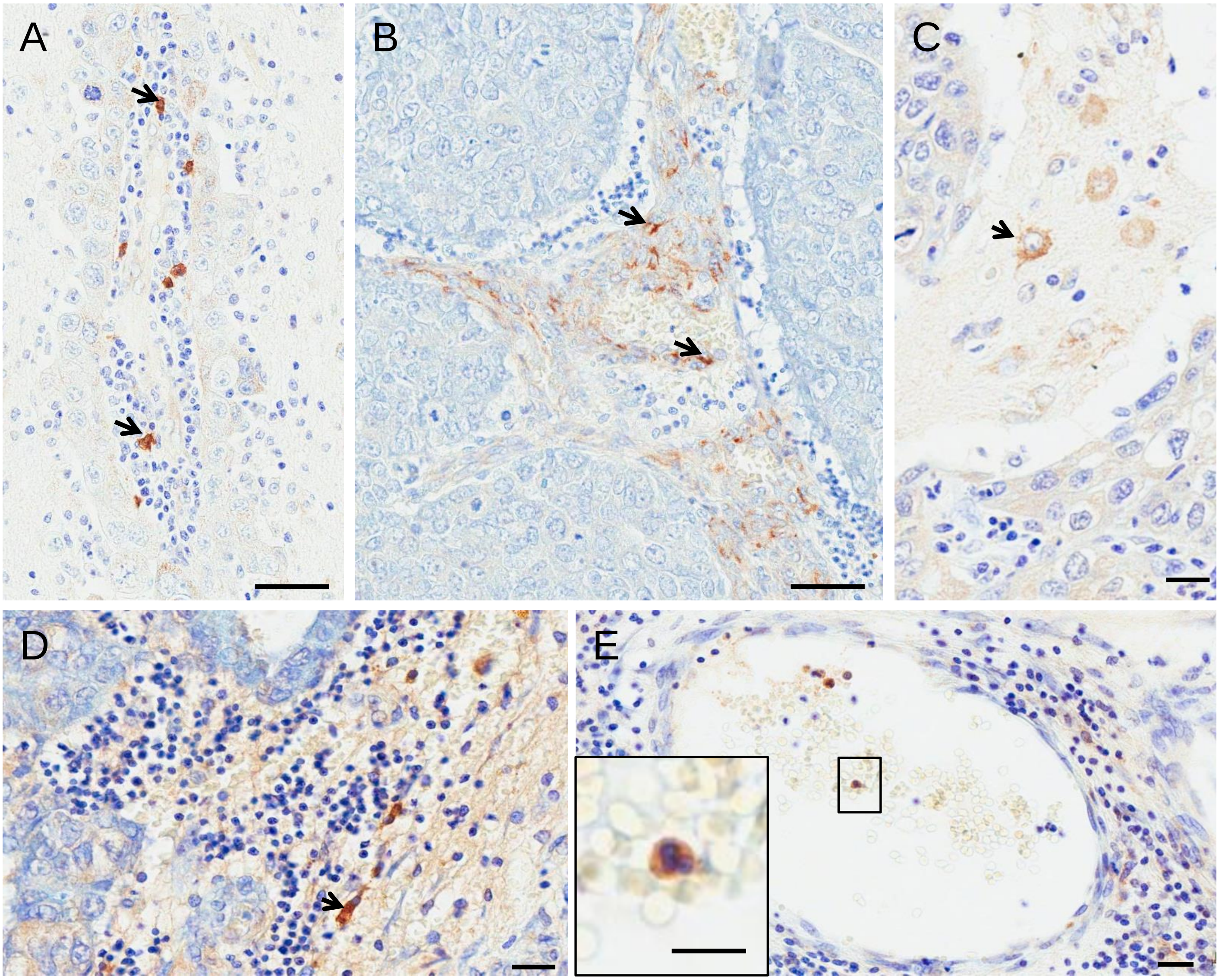

Supplementary figure 3. Non-endothelial TNF receptor expression in human brain metastasis. (A - B) TNFR1 staining on perivascular leukocytes and infiltrating cells within the metastasis. (C - E) TNFR2 staining on neurons near metastatic tissue, recruited leukocytes and intravascular neutrophils. Scale bars = 10 µm. Acronyms: TNF (tumor necrosis factor), TNFR1/2 (TNF receptor 1/2).

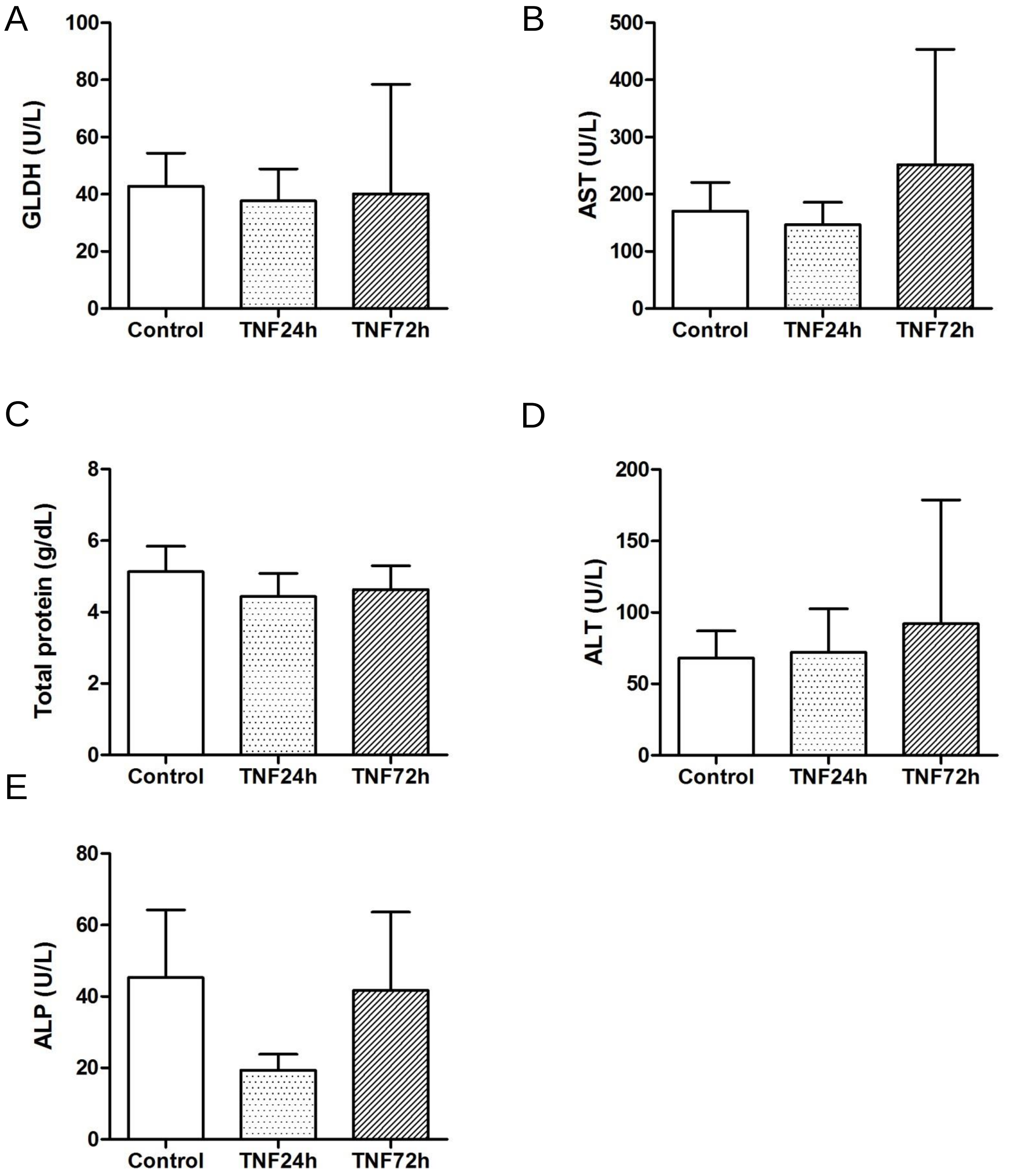

Supplementary figure 4. (A - E) Panel of liver function analyses of mouse plasma after systemic administration of 3 μg of rmTNF. No significant differences were found between any of groups for any of the liver function parameters measured, although a trend towards reduced ALP was evident 24h after TNF administration. This finding may indicate an acute suppression of liver protein synthesis following TNF administration, but this was no longer apparent by 72h. One animal appeared to show elevated ALT, AST and GLDH 72h after TNF administration, but the other 2 animals were well within normal ranges and no significant differences were found between the groups. Error bars represent standard deviation. Two-tailed 1-way ANOVA with Dunnet's multiple comparison post-test. Acronyms: rmTNF (recombinant mouse tumour necrosis factor), GLDH (glutamate dehydrogenase), AST (aspartate transaminase), ALT (alanine aminotransferase), ALP (alkaline phosphatase),

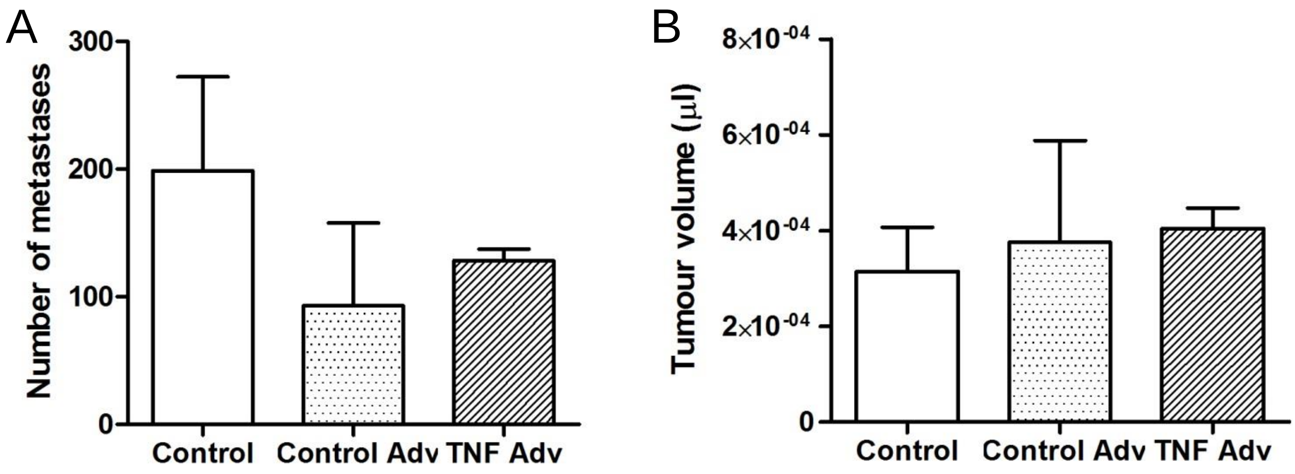

Supplementary figure 5. (A) Number and (B) volume of brain metastases in control mice and mice injected systemically with either a control null adenovirus or an adenovirus expressing membrane-bound TNF cDNA, which causes sustained systemic TNF expression. No significant differences were found between any of the groups for either number or volume of brain metastases. Error bars represent standard deviation. Two-tailed 1-way ANOVA with Dunnet's multiple comparison post-test. Acronyms: TNF (tumor necrosis factor).
